# Metabolic abnormalities exacerbate Sjögren’s syndrome by and is associated with increased the population of interleukin–17–producing cells in NOD/ShiLtJ mice

**DOI:** 10.1186/s12967-020-02343-7

**Published:** 2020-05-05

**Authors:** Sun-Hee Hwang, Jin-Sil Park, SeungCheon Yang, Kyung-Ah Jung, JeongWon Choi, Seung-Ki Kwok, Sung-Hwan Park, Mi-La Cho

**Affiliations:** 1 The Rheumatism Research Center, Catholic Research Institute of Medical Science, College of Medicine, The Catholic University of Korea, Seoul, 06591 Republic of Korea; 2 Divison of Rheumatology, Department of Internal Medicine, Seoul St. Mary’s Hospital, College of Medicine, The Catholic University of Korea, 222 Banpo-Daero, Seocho-gu, Seoul, 06591 Republic of Korea; 3 Department of Biomedicine & Health Sciences, College of Medicine, The Catholic University of Korea, Seoul, 06591 Republic of Korea; 4 Department of Medical Lifescience, College of Medicine, The Catholic University of Korea, Seoul, 06591 Republic of Korea; 5 Rheumatism Research Center, Catholic Institutes of Medical Science, The Catholic University of Korea, 222 Banpo-Daero, Seocho-gu, Seoul, 137-701 South Korea

**Keywords:** Sjögren’s syndrome, Type 1 diabetes, Interleukin-17, IL–17–producing T cell, IL–17–producing B cell

## Abstract

**Background:**

Sjögren’s syndrome (SS) is an autoimmune disease mediated by lymphocytic infiltration into exocrine glands, resulting in progressive lacrimal and salivary destruction and dysfunctional glandular secretion. Metabolic syndrome influences the immune system. To investigate its relationship with metabolic abnormalities, we evaluated the pathogenesis of SS and the immune cell populations in non-obese diabetic NOD/ShiLtJ mice with type 1 diabetes (T1D).

**Methods:**

To induce metabolic abnormalities, streptozotocin (STZ)—a glucosamine–nitrosourea compound that destroys pancreatic β cells, resulting in T1D—was injected into NOD/ShiLtJ mice. The blood glucose level was measured to evaluate induction of T1D. The severity of SS was assessed by determining the body weight, salivary flow rate, and histologic parameters. The expression levels of proinflammatory factors in the salivary glands, lacrimal gland, and spleen were quantified by real–time PCR. The populations of various T– and B–cell subtypes in the peripheral blood, spleen, and salivary glands were assessed by flow cytometry.

**Results:**

Induction of T1D in NOD/ShiLtJ mice increased both the severity of SS and the levels of proinflammatory cytokines in the salivary glands compared to the controls. Furthermore, the number of interleukin-17–producing immune cells in the peripheral blood, spleen, and salivary glands was increased in STZ- compared to vehicle-treated NOD/ShiLtJ mice.

**Conclusions:**

Metabolic abnormalities play an important role in the development of SS.

## Background

Sjögren’s syndrome (SS) is a systemic autoimmune disease characterized by infiltration of lymphocytes into the exocrine glands, inflammation, tissue damage, and dysfunctional glandular secretion. Destruction of the lacrimal and salivary glands, which typically occurs in patients with SS, results in ocular dryness (keratoconjunctivitis sicca) and oral dryness (xerostomia) [1]. Patients with SS often have extraglandular complications such as non-erosive polyarthritis, arthralgias, vasculitis, and chronic fatigue [2]. Furthermore, patients with SS have an increased incidence of progression to various non-Hodgkin lymphomas, which may influence the rate of morbidities [3].

The non-obese diabetic (NOD) mouse is not only a widely used model for diabetes mellitus type 1, but also recognized as an appropriate model to study autoimmune exocrinopathy prevalent in human SS patients. NOD mouse indicated the development of diabetes in 12-week-old NOD/ShiLtJ mice and an autoimmune exocrinopathy that shows significant similarities to SS becomes evident between 8 and 12 weeks [4]. “SS” in this model means cellular infiltration into the salivary gland or a change in salivary flow rate. In addition, the subsequent tissue specific immunological attack, antibody directed against the cell surface muscarinic/cholinergic receptors appears and increased cytokines in salivary gland [5–8].

The pathogenesis of SS is mediated by complex mechanisms involving infiltration by lymphocytes (mainly T and B cells) of target organs during a dysregulated adaptive immune response. In the T– and B–cell–containing ectopic lymphoid structures in the salivary and lacrimal glands, hyperactivated B cells produce autoantibodies; *e*.*g*., anti-SSA/Ro and -SSB/La, against small RNA molecules and rheumatoid factors [9, 10]. Activation of B cells by follicular helper T (Tfh) cells is crucial for the clonal selection and affinity maturation [11].

Immune cells are key mediators of chronic diseases associated with obesity or metabolic abnormalities. However, changes in metabolism also affect the immune system [12]. Metabolic abnormalities (cardiovascular risk factors, insulin resistance, and visceral obesity) lead to activation of immune cells in the adipose tissue, liver, and pancreas, and disrupt the co–ordination of the innate and adaptive immune responses [13–15]. In patients with SS, the prevalence and clinical significance of cardiovascular risk factors, which are associated with dyslipidemia, diabetes mellitus, and hyperuricemia, is higher than that in healthy control subjects [16, 17]. Furthermore, SS patients with metabolic syndrome have increased serum levels of leptin and interleukin (IL)-1β compared to those without metabolic syndrome [18]. However, little is known about the effects of metabolic abnormalities on the T and B lymphocytes that mediate the pathogenesis of SS.

We investigated the role of metabolic abnormalities induced by streptozotocin (STZ)—a glucosamine–nitrosourea compound that destroys pancreatic β cells, resulting in a type 1 diabetes (T1D) phenotype [19] —in the pathogenesis of SS in NOD/ShiLtJ mice. Induction of T1D in NOD/ShiLtJ mice increased the severity of SS, the levels of proinflammatory cytokines, and the number of IL–17–producing immune cells in the peripheral blood, spleen, and salivary glands. Also, STZ-induced metabolic abnormalities promoted the infiltration of IL–17–producing cells into the salivary glands.

## Methods

### Animals

Seven-week-old female NOD/ShiLtJ mice were purchased from Jackson Laboratories (Bar Harbor, ME) and were housed under specific pathogen-free conditions at the Catholic Research Institute of Medical Science, Catholic University of Korea. The mice were fed standard mouse chow and water. The procedures were approved by the Animal Research Ethics Committee of the Catholic University of Korea and conformed to the National Institutes of Health of the United States guidelines (Permit Number: CUMS- 2019-0001-01). All animals were treated and euthanized in accordance with the Guidelines on the Use and Care of Animals of the Catholic University of Korea. Surgery was performed under anesthesia with isoflurane, and every effort was made to minimize suffering. At the end of the study, the mice were euthanized in a CO_2_ chamber for sample collection.

### Injection of agents

To induce T1D, 10-week-old female NOD/ShiLtJ mice were fasted for 24 h and intraperitoneally injected with 180 mg/kg STZ (Sigma-Aldrich, St. Louis, MO) in 0.1 M Na-citrate buffer (pH 4.5), or with only 0.1 M citrate buffer. The blood glucose levels of the mice were determined using an Accu-Check™ Compact Glucometer on days 0, 3, 7, and 11 after STZ injection (Roche Diagnostics, Indianapolis, IN).

### Collection of saliva from NOD/ShiLtJ mice

Mice were anesthetized by inhalation of isoflurane (2%) and secretion of saliva was stimulated by intraperitoneal injection of pilocarpine (100 μg/mouse; Sigma-Aldrich). Ninety seconds later, saliva was collected using a micropipette from the oral cavity for 7 min. The volume of saliva collected was determined gravimetrically (μL/g/min). Saliva was stored at −70 °C until analysis.

### Histological assessment of salivary-gland inflammation

Parotid glands were fixed in 4% paraformaldehyde and embedded in paraffin; next, Sects. (5 μm thickness) were prepared and stained with hematoxylin and eosin. The degree of inflammation was quantified as described previously [20]. The sections were incubated with primary antibodies against IL-6, tumor necrosis factor-α (TNF-α), and IL-17 (Abcam, Cambridge, UK) overnight at 4 °C, and with a biotinylated secondary antibody (REAL™ EnVision™/horseradish peroxidase; Dako, Glostrup, Denmark) for 1 h at 4 °C. The color was developed using the chromogen diaminobenzidine (Thermo Scientific, Rockford, IL) and the sections were examined under a light microscope (Olympus, Tokyo, Japan). Cells positive for IL-6, TNF-α, or IL-17 were enumerated visually by four individuals on high-magnification images projected onto a screen; mean values are presented.

### Intracellular staining and flow cytometry

Cells were isolated from the spleen, salivary glands, and peripheral blood and stimulated with 25 ng/mL phorbol myristate acetate (Sigma-Aldrich) and 250 ng/mL ionomycin in the presence of GolgiStop (BD Biosciences, San Jose, CA) for 4 h. The cells were stained with anti-mouse CD4 peridin chlorophyll protein (PerCP) (clone RM4-5), anti-mouse C–X-C chemokine receptor type 5 (CXCR5)- PerCP-eFluor710 (clone SPRCL5) (eBioscience, San Diego, CA) and/or anti-mouse CD19 Phycoerythrin-Cy7 (clone 1D3) (BD Pharmingen, San Jose, CA) antibodies. Surface-labeled cells were permeabilized with Cytofix/Cytoperm solution (BD Pharmingen) and intracellular staining for IL-17 was performed using an anti-mouse IL-17–fluorescein isothiocyanate (clone eBio17B7) antibody (eBioscience, San Diego, CA). The samples were subjected to flow cytometry using a FACSCalibur (BD Pharmingen) and the data were analyzed using FlowJo software (Tree Star, Ashland, OR).

### Real-time polymerase chain reaction

A LightCycler 2.0 instrument (software version 4.0; Roche Diagnostics) and SensiFAST SYBR^®^ Hi–ROX (Bioline USA Inc., Taunton, MA) were used for polymerase chain reaction (PCR) amplification according to the manufacturer’s instructions. The following primers were used: IL-6, 5′-AAC GAT GCA CTT GCA GAA A-3′ (sense) and 5′-TCT GAA GGA CTC TGG CTT TGT C-3′ (antisense); RORγt, 5′–TGT CCT GGG CTA CCC TAC TG -3′ (sense), 5′- GTG CAG GAG TAG GCC ACA TT-3′ (antisense); IL-17, 5′- CCT CAA AGC TCA GCG TGT CC-3′ (sense), 5′- GAG CTC ACT TTT GCG CCA AG -3′ (antisense); TNF-α, 5′-ATG AGC ACA GAA AGC ATG ATC-3′ (sense) and 5′–TAC AGG CTT GTC ACT CGA ATT-3′ (antisense); and β-actin, 5′-GTA CGA CCA GAG GCA TAC AGG-3′ (sense) and 5′-GAT GAC GAT ATC GCT GCG CTG-3′ (antisense). The mRNA levels were normalized to that of β-actin.

### Statistical analysis

Statistical analyses were performed using GraphPad Prism (version 5 for Windows; GraphPad Software, San Diego, CA). Normally distributed continuous data were analyzed by parametric Student’s *t* test. Differences in mean values among groups were subjected to analysis of variance. Values are presented as means ± SD. Values of *P* < 0.05 (two-tailed) were considered indicative of significance.

## Results

### Induction of T1D in NOD/ShiLtJ mice increases the severity of SS

**Fig. 1:**
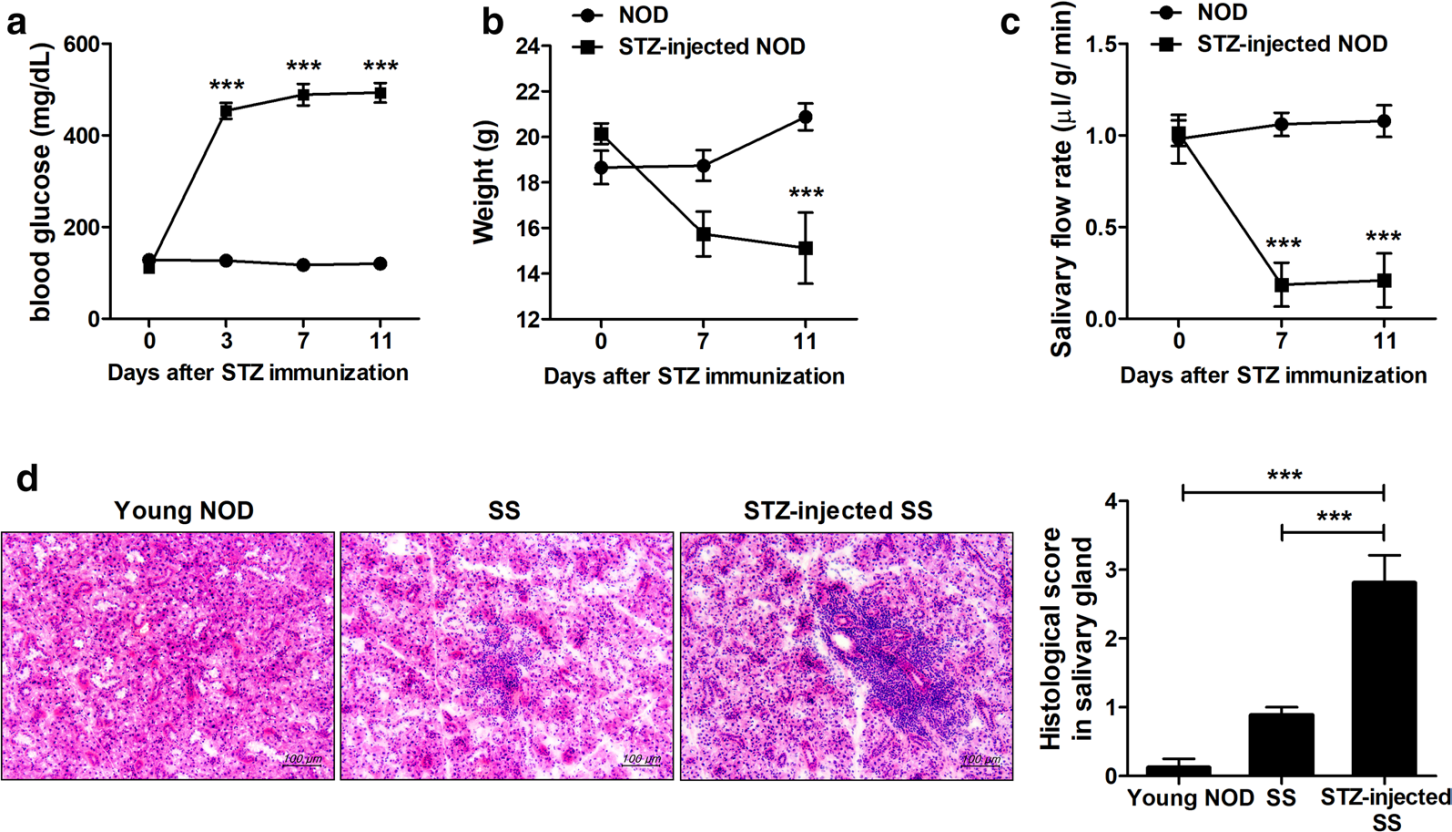
Streptozotocin (STZ) increases the severity of Sjögren’s syndrome (SS) in NOD/ShiLtJ mice. **a** After intraperitoneal injection of STZ (180 mg/kg) or vehicle into 10-week-old female NOD/ShiLtJ mice (*n* = 3 per group), the blood glucose level was determined using a glucometer. **b** Changes in body weight. **c** Salivary flow rates at 0, 7, and 11 days after injection of STZ. **d** Sections of parotid glands obtained 11 days after administration of STZ were stained with hematoxylin and eosin. Representative histological features and histologic grades are shown. ^***^P < 0.001 *vs*. vehicle–treated group. Data are mean ± SD

To evaluate the role of metabolic abnormalities in SS in vivo, 10-week-old female NOD/ShiLtJ mice were fasted for 24 h and STZ was administered intraperitoneally. Injection of STZ to mice and rats is associated with diabetes and weight loss [21–23]. Injection of STZ led to a rapid increase in the blood glucose level on day 3 and a decrease in body weight on day 7 compared to vehicle-treated NOD/ShiLtJ mice (Fig. 1a, b). Also, the salivary flow rate was markedly reduced in STZ-treated NOD/ShiLtJ mice compared to vehicle-treated NOD/ShiLtJ mice (*P* < 0.001) (Fig. 1c). Furthermore, infiltration of inflammatory cells into the salivary glands was exacerbated in STZ-treated NOD/ShiLtJ mice compared to vehicle-treated NOD/ShiLtJ mice (*P* < 0.001) (Fig. 1d). Therefore, metabolic abnormalities may contribute to the development of SS in NOD/ShiLtJ mice.

### Induction of T1D in NOD/ShiLtJ mice upregulates the levels of proinflammatory cytokines

**Fig. 2:**
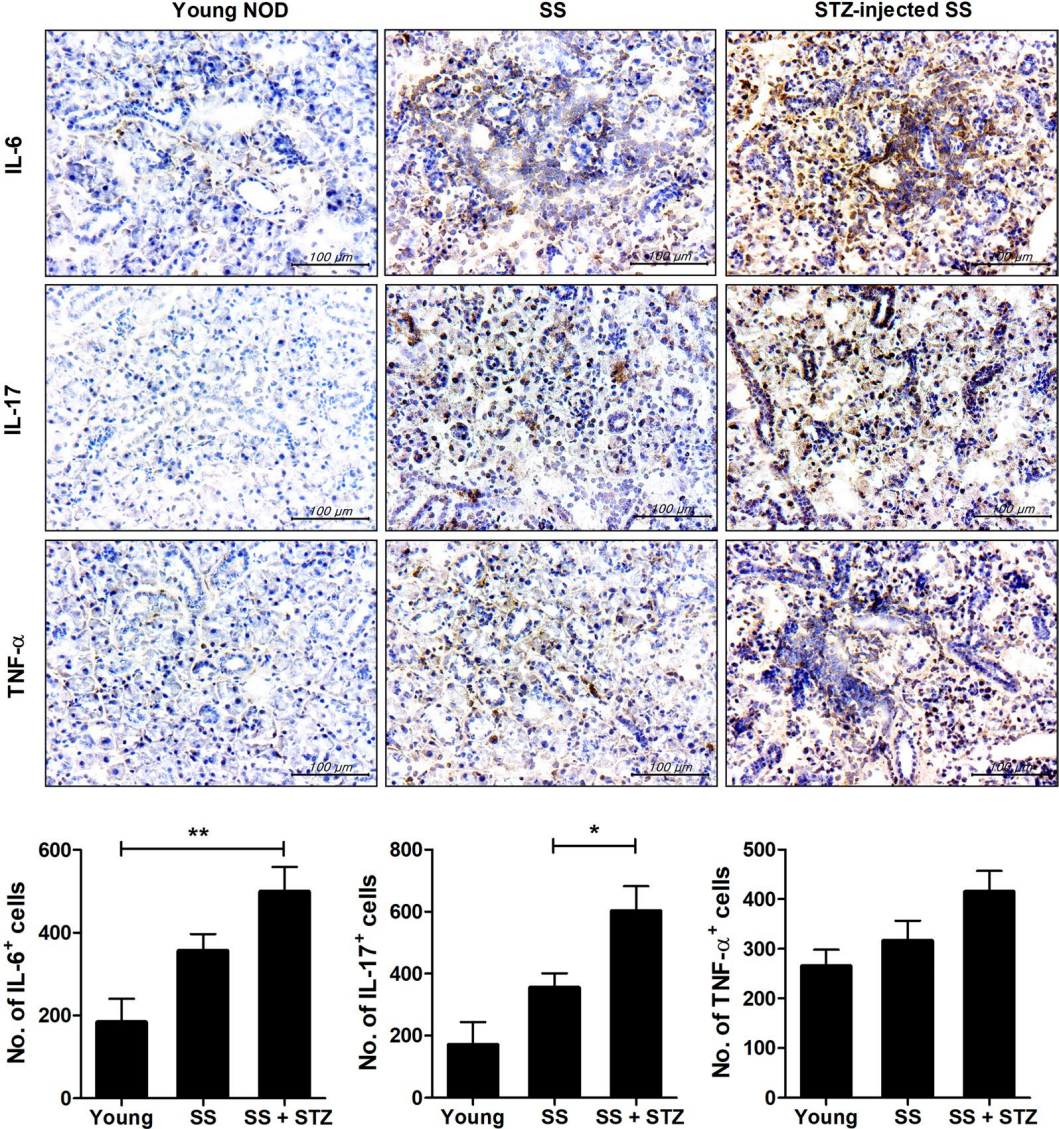
STZ increases the levels of proinflammatory cytokines in NOD/ShiLtJ mice. STZ (180 mg/kg) or vehicle was injected intraperitoneally into 10-week-old female NOD/ShiLtJ mice (*n* = 3 per group). Sections of parotid glands obtained 11 days after administration of STZ were stained with antibodies against interleukin (IL)-6, IL-17, and tumor necrosis factor-α (TNF-α). Representative histological features and numbers of antibody-positive cells are shown. Scale bar = 100 µm. ^*^P < 0.05 and ^**^P < 0.01 *vs*. vehicle-treated group. Data are mean ± SD

**Fig. 3:**
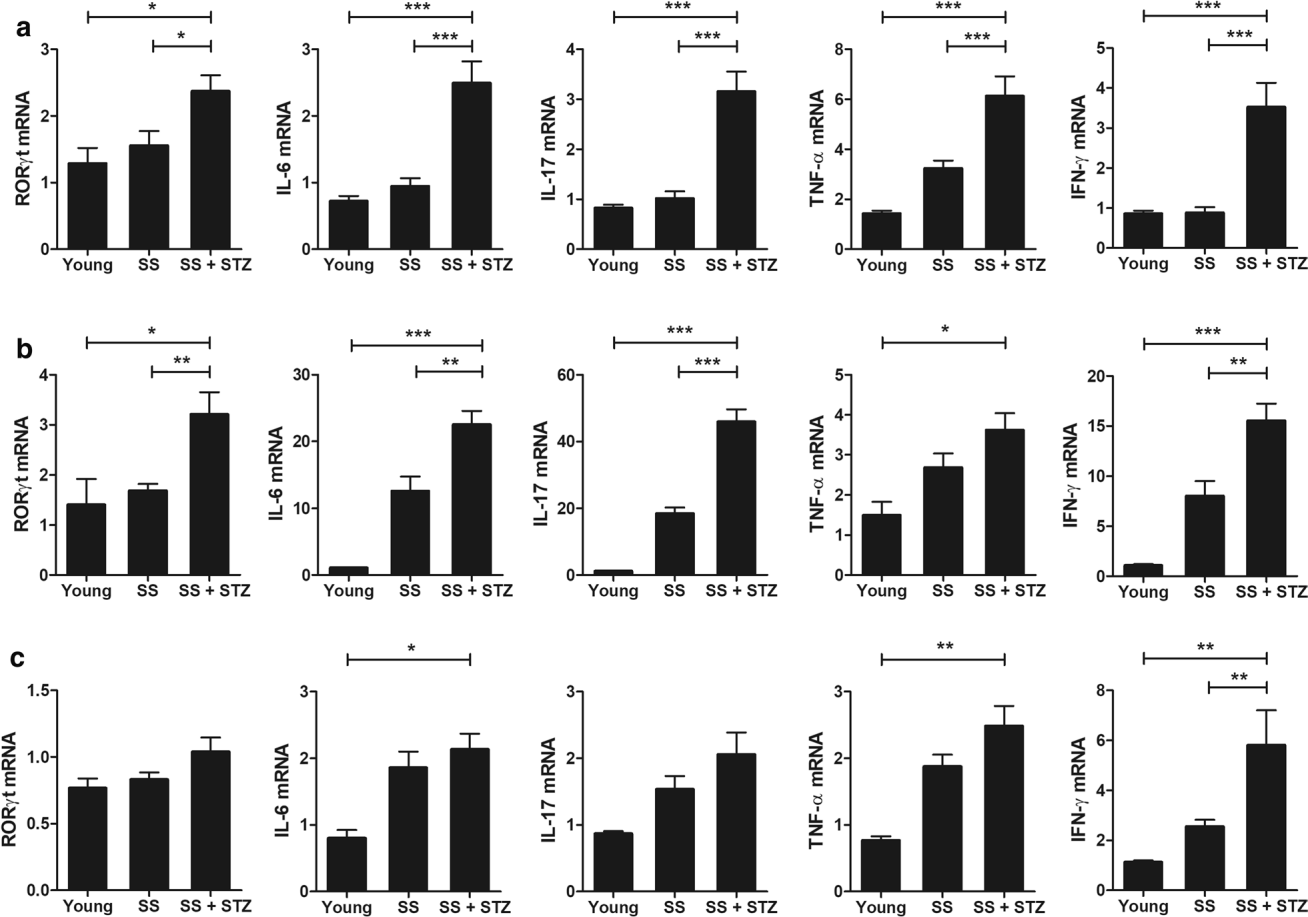
STZ increases the expression of proinflammatory cytokines in NOD/ShiLtJ mice. STZ (180 mg/kg) or vehicle was injected intraperitoneally into 10-week-old female NOD/ShiLtJ mice (*n* = 3 per group). RNA was extracted from the salivary glands (**a**), lacrimal glands (**b**), and spleens (**c**) of STZ– or vehicle-treated mice obtained 11 days after administration of STZ, and the mRNA levels of IL-6, RORγt, IL-17, IFN-γ, and TNF-α were analyzed by real-time polymerase chain reaction (PCR). ^*^P < 0.05, ^**^P < 0.01, and ^***^P < 0.001 *vs*. vehicle–treated group. Data are mean ± SD

To examine whether metabolic abnormalities affect the levels of proinflammatory cytokines, salivary-gland sections were immunohistochemically stained for IL-6, IL-17, and TNF-α. The number of IL-6 + and IL-17 + cells was significantly increased in the salivary glands of STZ-treated compared to vehicle-treated mice (*P* < 0.01 and *P* < 0.05, respectively) (Fig. 2a). The number of TNF-α + cells was non-significantly increased in the STZ-injected mice (Fig. 2). In addition, the mRNA levels of IL-6, RORγt, IL-17, IFN-γ, and TNF-α in cells from the salivary gland, lacrimal gland, and spleen were increased in STZ-treated compared to vehicle-treated mice (Fig. 3). Also, in STZ–treated mice, the mRNA levels of these proinflammatory cytokines were higher in the salivary glands and lacrimal glands than in the spleen. Therefore, induction of T1D in NOD/ShiLtJ mice promoted the secretion of proinflammatory cytokines.

### Metabolic abnormalities augment the number of IL–17–producing cells in NOD/ShiLtJ mice

**Fig. 4:**
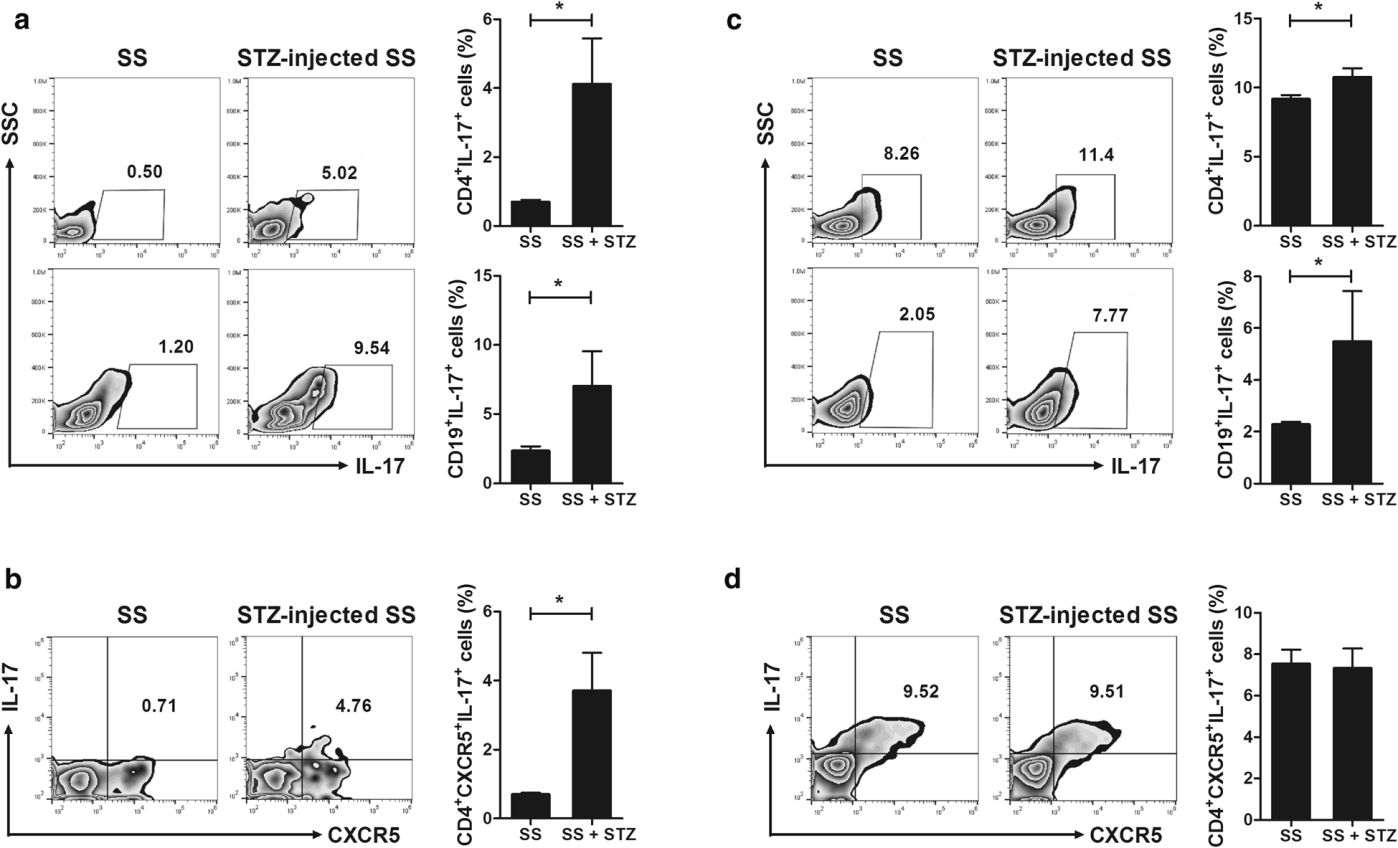
Induction of type 1 diabetes (T1D) increases the number of IL–17–producing cells in NOD/ShiLtJ mice. STZ (180 mg/kg) or vehicle was injected intraperitoneally into 10-week-old female NOD/ShiLtJ mice (*n* = 3 per group). **a, c** Peripheral blood cells (**a**) and splenocytes (**c**) obtained 11 days after administration of STZ were stimulated with PMA, ionomycin, and GolgiStop for 4 h and stained with antibodies against CD4^+^IL-17^+^ (Th17) and CD19^+^IL-17^+^ (B17) cells. **b, d** Peripheral blood cells (**b**) and splenocytes (**d**) obtained 11 days after administration of STZ were stimulated with PMA, ionomycin, and GolgiStop for 4 h and stained with antibodies against CD4^+^CXCR5^+^IL-17^+^ (Tfh17) cells, followed by analysis by flow cytometry. ^*^P < 0.05 *vs*. vehicle-treated group. Data are mean ± SD

To investigate the cell types related to the increased severity of SS in STZ-treated NOD/ShiLtJ mice, we analyzed the populations of T– and B–cell subtypes in peripheral blood and splenocytes. The numbers of Th17 cells and IL–17–producing B cells were significantly increased in the peripheral blood (Fig. 4a) and splenocytes (Fig. 4c) of STZ-treated NOD/ShiLtJ mice compared to those of vehicle-treated mice (*P* < 0.05). The number of CD4 + CXCR5 + IL-17 + Tfh17 cells in the peripheral blood was also significantly increased in NOD/ShiLtJ mice with T1D compared to vehicle-treated mice (*P* < 0.05) (Fig. 4b). However, there was no difference in the number of Tfh17 cells in splenocytes between the STZ- and vehicle-treated mice (Fig. 4d). Therefore, metabolic abnormalities may promote differentiation towards IL-17–producing cells.

### STZ-induced metabolic abnormalities increase infiltration of IL–17–producing cells in the salivary glands

**Fig. 5:**
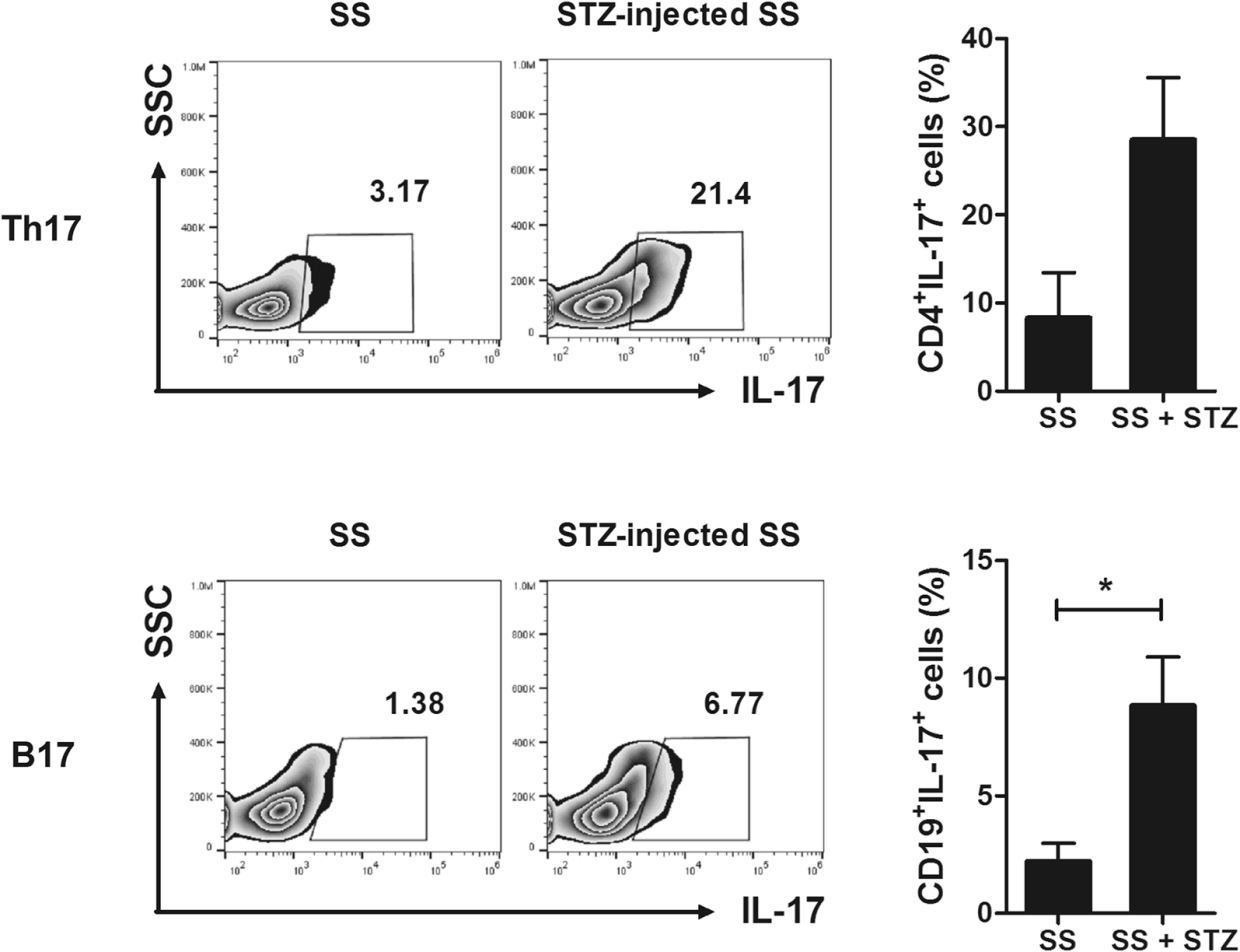
Induction of T1D promotes infiltration of IL–17–producing cells into the salivary glands of NOD/ShiLtJ mice. STZ (180 mg/kg) or vehicle was injected intraperitoneally into 10-week-old female NOD/ShiLtJ mice (*n* = 3 per group). Cells from salivary-gland tissue obtained 11 days after administration of STZ were stimulated with PMA, ionomycin, and GolgiStop for 4 h and stained with antibodies against CD4^+^IL-17^+^ (Th17) and CD19^+^IL-17^+^ (B17) cells, followed by analysis by flow cytometry. ^*^P < 0.05 *vs*. vehicle-treated group. Data are mean ± SD

We investigated whether the metabolic abnormalities induced by STZ promote infiltration of IL–17–producing cells into the salivary glands. Compared to vehicle-treated mice, the number of IL–17–producing T and B cells in the salivary glands of STZ-treated mice was dramatically increased (Fig. 5). Therefore, increased infiltration of IL–17–producing cells into the salivary glands results in destruction of salivary-gland tissue and an increase in the severity of SS.

## Discussion

We investigated the effect of metabolic abnormalities on T and B lymphocytes in NOD/ShiLtJ mice with SS and STZ-induced T1D. STZ resulted in a decrease in body weight and salivary flow rate, as well as in an increase in the infiltration of inflammatory cells into the salivary glands in NOD/ShiLtJ mice compared to vehicle-treated NOD/ShiLtJ mice. The number of IL–17–producing Th17, Tfh17, and B cells was increased in the peripheral blood and splenocytes from STZ- compared to vehicle-treated NOD/ShiLtJ mice. Furthermore, infiltration of IL–17–producing T and B cells into the salivary glands was increased in STZ-treated NOD/ShiLtJ mice. Therefore, metabolic abnormalities may contribute to the development of SS by increasing the population of IL–17–producing immune cells, as well as the infiltration of these cells into the salivary glands.

Metabolic syndrome, which comprises hypertension, diabetes, and obesity, is closely related to autoimmune diseases such as rheumatoid arthritis and systemic lupus erythematosus [24–27]. However, the clinical significance of metabolic abnormalities in patients with SS is unclear. Dyslipidemia is closely related to hyperuricemia and the clinical features of SS [16], and patients with SS have an increased prevalence of diabetes mellitus and hypertriglyceridemia compared to healthy control subjects [17]. Furthermore, patients with SS who also have metabolic syndrome have increased serum levels of leptin and IL-1β [18]. In this study, we evaluated the effect of metabolic abnormalities on the development of SS. Induction of T1D in NOD/ShiLtJ mice greatly decreased the salivary flow rate and increased the degree of inflammation in the salivary glands. Also, the number of IL–17–producing cells in peripheral blood and splenocytes was increased in STZ- compared to vehicle-treated NOD/ShiLtJ mice. In particular, the increase in the population of IL-17–producing B cells can be used as an indicator of lymphoma in SS; thus, it is an important guide for SS treatment strategies [3]. These results clarify the effect of metabolic abnormalities on the immune cells involved in the development of SS. Further studies should aim to identify the molecular mechanism by which metabolic abnormalities increase IL-17 production by T and B cells.

IL-17 is a proinflammatory cytokine involved in the pathogenesis of various autoimmune diseases, including rheumatoid arthritis, systemic lupus erythematosus, and psoriasis [28]. IL-17 triggers the production of proinflammatory factors, including IL-21, IL-6, CXCL8/IL-8, and CCL2/MIP–3a, which promotes lymphocyte recruitment, activation, and migration to target tissues [29–31]. Although IL-17 is mainly secreted by Th17 cells, it is also produced by Tfh and B cells [32–34]. The expression level of IL-17 is high in salivary gland tissues from C57BL/6.NOD-*Aec*1*Aec*2 mice and SS patients [35], and IL-17–expressing CD4 + T cells are present in the salivary glands of SS patients [36]. In addition, IL-17–deficient salivary gland protein-immunized SS mice exhibit reduced susceptibility to SS, but adoptive transfer of Th17 cells into IL-17–knockout mice induces SS by mimicking immunization with salivary gland proteins [37]. Diabetic children have higher levels of IL-17 and salivary mediators in saliva than nondiabetic children [38]. Therefore, IL-17 is related to the severity of SS. Metabolic syndrome reportedly exacerbates IL-17–mediated inflammatory reactions. The level of IL-17 in the periapical, hepatic, and renal regions was increased in apical periodontitis with STZ-induced diabetes compared to apical periodontitis [39]. Furthermore, administration of an IL-17 blocker reduces the pathogenicity of the oral microbiota in diabetic mice [40], and STZ-treated IL-17–knockout mice show decreased hyperglycemia and insulitis compared to control mice [41]. Adiponectin secreted from white adipose tissue promotes the development of arthritis by increasing the number of Th17 cells and the expression of receptor activator of nuclear factor-κB ligand in the joint tissues of mice with collagen-induced arthritis [42]. In addition, obesity promotes the differentiation of Th17 cells by increasing the expression of lipid kinase in CD4 T cells [43]. These previous reports support our hypothesis that metabolic abnormalities promote the development of IL-17–mediated SS. Further study of the mechanism(s) by which metabolic abnormalities promote the pathogenesis of SS is needed.

## Conclusion

To our knowledge, this is the first report showing that metabolic syndrome exacerbates SS by increasing both the number of IL–17–producing immune cells and their infiltration into the salivary glands. Our findings suggest that metabolic disorders play an important role in the development of SS.

## Data Availability

All data are available in the manuscript or upon request to the authors.

## Abbreviations

SS: Sjögren’s syndrome
Tfh: Follicular helper T
IL: Interleukin
STZ: Streptozotocin
T1D: Type 1 diabetes
TNF-α: Tumor necrosis factor-α
CXCR5: C X-C chemokine receptor type 5
FITC: Fluorescein isothiocyanate
PCR: Polymerase chain reaction
